# Collateral state and the effect of endovascular reperfusion therapy on clinical outcome in ischemic stroke patients

**DOI:** 10.1002/brb3.513

**Published:** 2016-06-17

**Authors:** Johannes C. Gerber, Marketa Petrova, Pawel Krukowski, Matthias Kuhn, Andrij Abramyuk, Ulf Bodechtel, Imanuel Dzialowski, Kay Engellandt, Hagen Kitzler, Lars‐Peder Pallesen, Hauke Schneider, Ruediger von Kummer, Volker Puetz, Jennifer Linn

**Affiliations:** ^1^ NeuroradiologyUniversity Hospital Carl Gustav CarusDresdenGermany; ^2^ RadiologyUniversity Hospital Carl Gustav CarusDresdenGermany; ^3^ Institute of Medical Informatics and BiometryMedizinische Fakultät Carl Gustav CarusTechnische UniversitätDresdenGermany; ^4^ NeurologyUniversity Hospital Carl Gustav CarusDresdenGermany; ^5^ NeurologyElblandklinikumMeissenGermany

**Keywords:** Angiography, endovascular treatment, ischemic stroke, leptomeningeal collaterals

## Abstract

**Purpose:**

Clinically successful endovascular therapy (EVT) in ischemic stroke requires reliable noninvasive pretherapeutic selection criteria. We investigated the association of imaging parameters including CT angiographic collaterals and degree of reperfusion with clinical outcome after EVT.

**Methods:**

In our database, we identified 93 patients with large vessel occlusion in the anterior circulation treated with EVT. Besides clinical data, we assessed the baseline Alberta Stroke Program Early CT score (ASPECTS) on noncontrast CT (NCCT) and CT angiography (CTA) source images, collaterals (CT‐CS) and clot burden score (CBS) on CTA and the degree of reperfusion after EVT on angiography. Three readers, blinded to clinical information, evaluated the images in consensus. Data‐driven multivariable ordinal regression analysis identified predictors of good outcome after 90 days as measured with the modified Rankin Scale.

**Results:**

Successful angiographic reperfusion (OR 26.50; 95%‐CI 9.33–83.61) and good collaterals (OR 9.69; 95%‐CI 2.28–59.27) were independent predictors of favorable outcome along with female sex (OR 0.35; 95%‐CI 0.14–0.85), younger age (OR 0.88; 95%‐CI 0.83–0.92) and higher NCCT ASPECTS (OR 2.54; 95%‐CI 1.01–6.63). Outcome was best in patients with good collaterals and successful reperfusion, but there was no statistical interaction between collaterals and reperfusion.

**Conclusions:**

CTA‐collateral status was the strongest pretherapeutic predictor of favorable outcome in ischemic stroke patients treated with EVT. CTA‐collaterals are thus well suited for patient selection in EVT. However, the independent effect of reperfusion on outcome tended to be stronger than that of CTA‐collaterals.

## Introduction

Ischemic stroke is frequently caused by an acute vessel occlusion leading to brain parenchymal oligemia or ischemia. Pial collaterals have been recognized as a prognostic factor for favorable clinical outcome in ischemic stroke patients (Liebeskind 2003; McVerry et al. 2012), as they can temporarily compensate regional ischemia to a level that allows tissue survival (Astrup et al. 1979). Pial collaterals can best be visualized by digital subtraction angiography (DSA; Von Kummer et al. 1995; Kucinski et al. 2003). However, noninvasive imaging‐based selection criteria are needed, to quickly decide for or against endovascular therapy (EVT) in ischemic stroke. To that aim, the assessment of collateral status on pretreatment CT angiography (CTA) has been proposed (Schramm et al. 2002; Tan et al. 2007; Miteff et al. 2009). In contrast to DSA (Higashida et al. 2003), no generally accepted grading system exists so far for the assessment of collaterals on CTA (McVerry et al. 2012).

Despite lacking standardization in their grading, good collaterals on CTA have a positive prognostic effect on clinical outcome in ischemic stroke patients treated with intravenous thrombolysis (IVT; Miteff et al. 2009; Lima et al. 2010; Menon et al. 2011). Besides collateral status, recanalization is strongly associated with improved functional outcomes and reduced mortality (Rha and Saver 2007), more specifically, substantial angiographic reperfusion seems to be the best predictor for independence after 3 months (Yoo et al. 2013). The combined effect of reperfusion and pretreatment CTA‐collateral status regarding clinical outcome has been evaluated in ischemic stroke patients treated with IVT (Miteff et al. 2009) and EVT with ambiguous results (Nambiar et al. 2013; Seeta Ramaiah et al. 2014).

To clarify the latter, we investigated the effect and association of CTA‐collaterals and reperfusion on outcome in patients with ischemic stroke due to large vessel occlusion in the anterior circulation having been treated with EVT. Secondarily, we analyzed various clinical and imaging outcome predictors.

## Materials and Methods

### Study population

We retrospectively analyzed 180 patients who received EVT for ischemic stroke in the anterior circulation at our institution from January 2010 to December 2012. According to our institutional protocol, EVT was performed if (1) an intracranial hemorrhage was excluded on noncontrast CT (NCCT), (2) the extent of early ischemic changes on NCCT was smaller than 1/3 of the middle cerebral artery territory, and (3) an intracranial large vessel occlusion was detectable on CTA. No time constraints were set for the delay from symptom onset to start of EVT. Prior to EVT, IVT was administered to eligible patients.

We included patients from our stroke database meeting the following criteria: (1) NCCT/CTA and follow‐up imaging available and performed at our institution, (2) occlusion of the intracranial internal carotid artery (ICA), or the middle cerebral artery (MCA, either M1 or M2 segment) confirmed by CTA, (3) EVT performed. Exclusion criteria were severe concomitant disease with influence on outcome.

The study was approved by our ethics committee (EK210072012). Informed consent prior to study inclusion was waived due to the retrospective nature of the study.

### Assessment of clinical course and functional outcome

We reviewed the medical records and documented the patient characteristics listed in Table 1. Functional outcome was assessed with the modified Rankin Scale (mRS) score at 90 days after treatment. The mRS scores were obtained from the medical records or the rehabilitation discharge summaries. If these were not available or did not contain outcome information, a physician trained in mRS scoring, blinded to the imaging results, contacted the patients or families to determine the functional status of the patient with a structured interview (Van Swieten et al. 1988).

**Table 1:** Clinical baseline and treatment characteristics

	*n*	%	Median	Range	IQR
Age			69	36–90	13.5
Women/Men	45/48	48%/52%			
Pretreatment NIHSS score			17	1–34	8
Comorbidities					
Atrial fibrillation	45	48%			
Arterial hypertension	72	77%			
Diabetes	30	32%			
Dyslipidemia	52	56%			
Previous myocardial infarction	5	5%			
Previous stroke	8	9%			
IVT	56	60%			
EVT with general anesthesia	82	88%			
Endovascular treatment					
Carotid artery stent	23	25%			
Aspiration thrombectomy (total)	47	51%			
With IA tPA	17				
Stent Retriever (total)	36	39%			
With distal aspiration	34				
With IA tPA	8				
Intra‐arterial tPA alone	3	3%			
No EVT					
No access to occlusion	3	3%			
Spontaneous recanalization	4	4%			
Serious					
Vessel perforation	4	4%			
Adverse					
Device breakage	2	2%			
Events					
Contralateral infarction (non‐EVT related)	1	1%			
Onset‐to‐CT time (minutes)			115		149
CT‐to‐treatment (angiography) time (minutes)			114		60
Onset‐to‐treatment time					
IVT			119		73
Angiography			252		154
90‐day favorable outcome (mRS ≤ 2)	29	31%			
90‐day mortality (mRS 6)	19	20%			

IVT, Intravenous thrombolysis; EVT, Endovascular treatment; mRS, modified Rankin Scale.

The endpoints were the score on the mRS 90 days after EVT (mRS ≤ 2 being favorable outcome), death at 90 days (mRS 6), and intracranial hemorrhage on follow‐up imaging (ECASS II classification; Larrue et al. 2001; Trouillas and von Kummer 2006).

### Imaging acquisition and postprocessing

CT imaging was performed on a Siemens SOMATOM Sensation 16 (Siemens Healthcare, Erlangen, Germany). At baseline NCCT was acquired (contiguous nonhelical axial slices with 1.5 and 6 mm thickness, oriented parallel to the orbitomeatal line) immediately followed by CTA from the aortic arch to the vertex. CTA was obtained after an IV injection (4 mL/sec) of 100 mL contrast followed by a saline bolus of 50 mL. Dynamic bolus‐tracking technique optimized image acquisition at peak contrast arrival. The CTA‐scan had a collimation of 16 × 0.75 mm, a rotational speed of 2 per second, a pitch of 1.25 and a scan length of 240–300 mm.

CT angiography data were reformatted as (1) 2 mm multiplanar reconstructions (MPR; window width: 200 HE, window center: 800 HE) for assessment of the vessel lumen, (2) axial CTA source images reconstructed to match the NCCT and displayed with window center and level settings where maximum contrast between normal and ischemic tissue was visible; and (3) maximum intensity projections (MIP) in overlapping 7 mm axial slabs.

### Endovascular treatment

We performed DSA and EVT on a biplane angiography system (Philips Allura Xper, Philips, Eindhoven, Netherlands). For the intervention, patients were either in general anesthesia or under conscious sedation. For EVT, we used three techniques alone or in combination: (1) intra‐arterial tPA via microcatheter, (2) aspiration thrombectomy (Penumbra System; Penumbra Inc, Alameda, CA), and (3) mechanical thrombectomy with stent retrievers (APERIO, Acandis GmbH, Pforzheim, Germany; pREset, phenox GmbH, Bochum, Germany; Separator 3D, Penumbra Inc.; Solitaire FR, ev3 Neurovascular, Irvine, CA). An additional occlusion of the extracranial ICA was treated first with stenting and angioplasty. EVT was halted when successful reperfusion defined as modified Treatment in Cerebral Ischemia (mTICI) 2b or 3 (Yoo et al. 2013) was confirmed or if reperfusion could not be achieved within 2 h (Broderick et al. 2013). All interventions were registered with an external quality control program from July 2010 onwards.

Follow‐up imaging was performed 24 ± 12 h after baseline CT either as NCCT or MRI (including diffusion‐weighted imaging [DWI] sequences and blood‐sensitive sequences [SWI, T2*]).

### Analysis of imaging data sets

One stroke neurologist and two neuroradiologists analyzed all CT/MRI images and three neuroradiologists read the DSA. Imaging parameters were read independently and in consensus using established scores as described below. Readers were blinded to clinical information. A different patient order during each reading session excluded recall bias. To assess inter‐observer agreement, the readers independently evaluated the images of 20 randomly selected patients.

### Analysis of baseline NCCT and CTA data sets

We searched the baseline NCCT for early ischemic changes and defined the Alberta Stroke Program Early CT score (NCCT ASPECTS; Barber et al. 2000). Any intra‐ or extracranial vascular occlusion was determined on CTA. The most proximal intracranial occlusion was designated the target arterial lesion (Zaidat et al. 2013). The clot burden score (CBS; Puetz et al. 2008) was assessed on CTA. CTA source images (CTA‐SI) were used to grade the CTA‐SI ASPECTS (Bhatia et al. 2011). We rated pial collaterals on CTA using a previously described score (CT‐CS; Tan et al. 2007, 2009).

### Angiographic assessment

On DSA, we assessed intra‐ or extracranial vessel occlusions. After EVT, we graded reperfusion for the target arterial lesion, using the mTICI score (Zaidat et al. 2013).

### Follow‐up imaging

We rated follow‐up images (NCCT or MRI‐DWI) for final infarct extent using ASPECTS, and graded hemorrhages according to the ECASS II classification (Larrue et al. 2001; Trouillas and von Kummer 2006). Only PH2 was defined as a clinically significant hemorrhage (Berger et al. 2001).

### Statistical analysis

Baseline population data are reported using standard descriptive statistics. We estimated the effect of the clinical and radiological variables on outcome with an ordinal logistic regression (proportional odds) model (Bender and Benner 2000; Saver 2007), which considers the whole range of the mRS. Variable selection was data driven. Therefore, we unselectively entered the following variables into the analysis: age, sex, diabetes, atrial fibrillation, previous stroke or myocardial infarction, arterial hypertension, hypercholesterolemia, admission NIHSS, NCCT ASPECTS, CTA‐SI ASPECTS, CT‐CS, CBS, target arterial lesion, IVT, time to EVT, type of anesthesia, type of EVT (thrombus aspiration alone; stent retriever in combination with direct aspiration; stent retriever alone), and reperfusion (mTICI). We dichotomized the variables reperfusion (mTICI 0/1/2a | 2b/3), clot burden score (CBS ≤5 | >5), NCCT ASPECTS and CTA‐SI ASPECTS (≤7 | >7) into two groups, favoring poor and good outcome (Hill et al. 2003; Puetz et al. 2008; Menon et al. 2011; Yoo et al. 2013). Due to a lack of published data, we dichotomized the variable CT‐CS based on a nonparametric Kruskal–Wallis one‐way analysis of variance.

We visualized the predicted cumulative probabilities for the final proportional odds model. Validity and discriminatory power of the model were tested.

To assess inter‐rater agreement of the imaging parameters, we used Kendall's coefficient of concordance W, which can handle ordinal scaled measurements from more than two raters. For the interpretation of Kendall's *W* the same scale as for Fleiss' kappa can be used as a nonevidenced guideline: 0.21–0.40 fair agreement; 0.41–0.60 moderate; 0.61–0.80 substantial; 0.81–1.00 almost perfect agreement.

All hypothesis tests were two‐tailed, with a value of *P* < 0.05 considered statistically significant.

We used the software R Project for Statistical Computing (R Development Core Team, 2015; RRID:SCR_001905) for analysis.

## Results

### Baseline population characteristics

**Figure 1:**
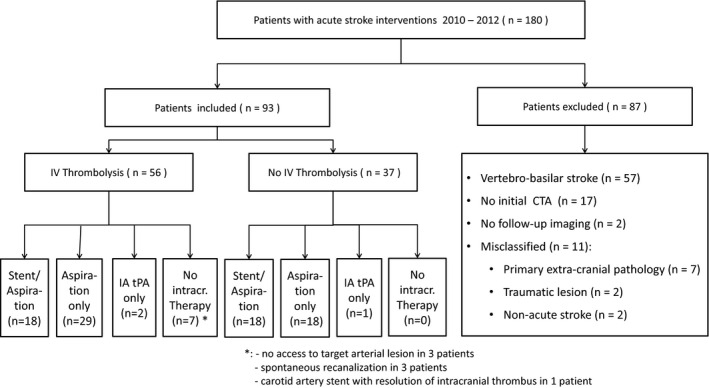
Patient flow chart.

During the observation period, we treated 180 ischemic stroke patients with EVT. Of these, we had to exclude 87 patients, leaving 93 patients (48 men; 52%) for analysis (Fig. 1). Their median baseline NIHSS score was 17 (range 1–34) and median age was 69 years (range 36–90; Table 1). Intravenous thrombolysis (IVT) was administered in 56 (60%) patients prior to EVT (bridging therapy). The mean onset to IVT treatment time was 125 min (median 119 min, IQR 73 min).

The location of the target arterial lesion and concomitant extracranial ICA disease according to the CTA are given in Table 2.

**Table 2:** Imaging characteristics

	*n*	%	Median	Range	IQR	Inter‐rater agreement (Kendall's *W*)
NCCT ASPECTS	93		7	3–10	2	0.784
CTA‐SI ASPECTS	93		5	0–10	3	0.907
CBS	93		5	0–10	4	
Target arterial lesion (on CTA)						
Infraclinoidal ICA	35	37%				
Supraclinoidal ICA	14	15%				
Proximal M1‐segment	20	22%				
Distal M1‐segment	20	22%				
M2‐segment	4	4%				
Collaterals (CT‐CS)						
0	5	5%				0.752
1	8	9%				
2	52	56%				
3	28	30%				
Reperfusion (mTICI)						
0	9	10%				0.396
1	5	5%				
2a	25	27%				
2b	46	49%				
3	8	9%				
Follow‐up imaging						
ASPECTS	93		5	0–10	5	
Hemorrhage						
HI 1	30	32%				
HI 2	14	15%				
PH 1	4	4%				
PH 2	1	1%				
SAH	9	10%				

CBS, Clot Burden Score; CTA, CT angiography; ICA, internal carotid artery.

### Endovascular therapy and clinical outcome

Prior to intracranial recanalization 23 patients (25%; 20 men) received carotid artery stenting. For recanalization of the intracranial occlusion, we performed aspiration thrombectomy alone in 47 (51%) patients, in 36 (39%) patients we used a stent‐retriever with (*n* = 34) or without aspiration at the occlusion (*n* = 2). In 3 (3%) patients, we administered intra‐arterial tPA as the only endovascular therapy and in 25 patients along with other treatments.

We treated eleven patients in conscious sedation, while 82 (88%) patients received general anesthesia during EVT.

We experienced the following complications: subarachnoid hemorrhage in 4 (4%) patients, device breakage in 2 (2%) patients, and a contralateral large infarction in one patient. Follow‐up imaging revealed hemorrhagic infarction type 1 (HI 1) in 30 (32%) patients, and HI 2 in 14 (15%) patients, while four (4%) patients had PH 1, and one patient developed a large intracerebral hemorrhage (PH 2). At 3 months, 29 (31%) patients had a favorable outcome (mRS ≤2), 45 (49%) patients had an unfavorable outcome (mRS 3–5), and 19 (20%) patients had died (mRS 6; Table 1).

### Imaging results

The imaging results are summarized in Table 2. The median baseline NCCT ASPECTS was 7, the median CTA‐SI ASPECTS was 5 and the median clot burden score (CBS) was 5.

On baseline CTA, five (5%) patients had no collaterals (CT‐CS 0), a CT‐CS of 1 was present in eight (9%) patients, a CT‐CS of 2 was present in 52 (56%) patients, and 28 (30%) patients had a CT‐CS of 3. A poorer collateral grade was associated with a lower CBS (Spearman *ρ*: 0.28; *P* < 0.01). No association was found between the site of the intracranial occlusion and the collateral grade (CT‐CS). However, patients with a poorer collateral grade tended to have more proximal occlusions: all patients with CT‐CS 0 (*n* = 5) had an intracranial ICA occlusion. We dichotomized patients with a CT‐CS of 0 and 1 to the poor collateral group (*n* = 13) and those with a CT‐CS of 2 and 3 to the good collateral group (*n* = 80) based on an analysis of variance showing better outcomes for patients with a CT‐CS of ≥2 (*P* < 0.01). The two groups did not differ regarding other clinical variables.

We achieved substantial angiographic reperfusion (mTICI 2b/3) in 54 patients (58%).

There was almost perfect inter‐rater agreement for CTA SI‐ASPECTS with Kendall's *W* of 0.907; for NCCT ASPECTS: 0.784 (substantial agreement); for CT‐CS: 0.752 (substantial agreement); and for mTICI 0.396 (fair agreement).

### Predictors of functional outcome

In the final ordinal regression model successful reperfusion (*P* < 0.001), good collaterals (*P* = 0.001), younger age (*P* < 0.01), female sex (*P* < 0.02), and a good initial NCCT ASPECTS (*P* < 0.05) were independently associated with better functional outcome (Table 3). Shorter onset to treatment time and IVT showed a trend for better outcome. No statistically significant interactions were found by the model.

**Table 3:** Summary of the final ordinal regression model

Factor	OR	Lower CI (0.95) OR	Upper CI (0.95) OR	*P* value
Sex	0.35	0.14	0.85	0.021
Age	0.88	0.83	0.92	0.000
IVT	0.44	0.16	1.16	0.098
NCCT‐ASPECTS	2.54	1.01	6.63	0.048
CT‐CS	9.69	2.28	59.27	0.001
mTICI	26.50	9.33	83.61	0.000
OTT (EVT)	0.39	0.12	1.19	0.097

EVT, Endovascular treatment; IVT, Intravenous thrombolysis; mTICI, modified Treatment in Cerebral Ischemia; OR, Odds ratio.

Odds ratios of the different variables for a shift to better clinical outcome are listed. The given variables survived computational variable reduction and were evaluated in the final regression. The OR for age is given per 1‐year increase. All other variables are dichotomized as described, and *P*‐values resulted from a likelihood ratio test.

The ordinal regression seemed valid as no major deviations from the proportional odds assumption were detectable. The discriminatory ability of the final model was sufficiently strong with a bias‐corrected Somer's *D* of 0.67.

### Collateral status and functional outcome

Only one patient of the poor collateral group (in total *n* = 13) had a good outcome (mRS ≤ 2). In this group, an unfavorable outcome was seen in two patients (15%, 95%‐CI: 4–42%), but most patients died (*n* = 10, 77%, 95%‐CI: 50–92%). Of the patients with a good CT‐CS (*n* = 80), 28 had good outcomes (mRS ≤ 2; 35%, 95%‐CI: 25–46%), 43 had unfavorable outcomes (54%, 95%‐CI: 43–64%), and nine patients died (11%, 95%‐CI: 6–20%). In general, patients with a poor CT‐CS tended to be less likely to have a good outcome (OR: 0.15; 95%‐CI: 0.02–1.25) and were more likely to die (OR: 26.29; 95%‐CI: 6.08–113.78).

### Association of collateral status and reperfusion on functional outcome

**Figure 2:**
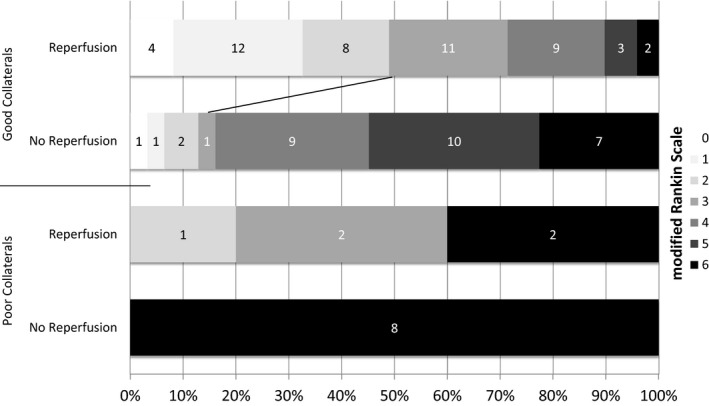
Ninety‐day clinical outcome as assessed by the modified Rankin Scale score (*n* = 93); stratified according to dichotomized collateral grade (Poor Collaterals: CT‐CS 0/1 versus Good Collaterals: CT‐CS 2/3) and dichotomized angiographic reperfusion (No‐Reperfusion: mTICI 0/1/2a versus Reperfusion: mTICI 2b/3).

**Figure 3:**
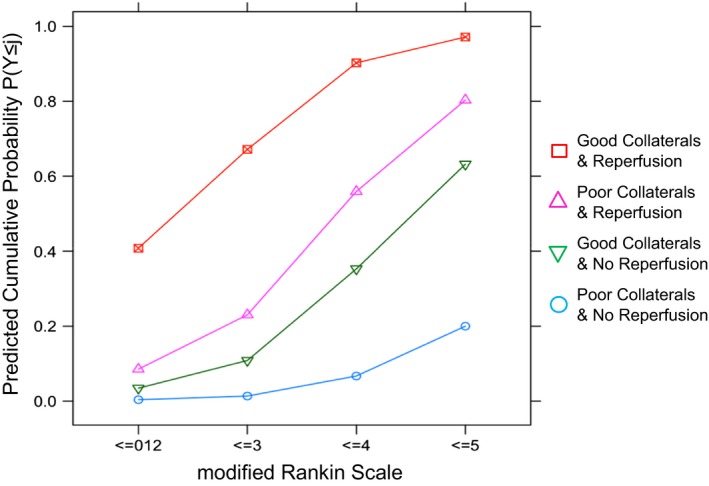
Predicted 90 day clinical outcome by collateral status and reperfusion. The figure shows predicted cumulative probabilities of outcome with regard to collaterals and reperfusion. The probabilities are calculated for a 70‐year‐old patient averaged over sex. The *X*‐axis denotes the mRS‐categories (mRS 0–2 are summarized). All curves converge at the intersection *P* = 1 and mRS 6, not shown. The probability for fatal outcome is the distance from the last data point to this intersection.

The combined effect of collateral status (CT‐CS) and reperfusion (mTICI) on functional outcome (mRS) is given in Figure 2. Of the patients with poor collaterals and no reperfusion (*n* = 8), all died. In patients with poor collaterals, successful reperfusion (*n* = 5) was associated with favorable clinical outcome in only one patient (20%, 95%‐CI: 3–62%), with unfavorable outcome in two patients (40%, 95% ‐CI: 12–77%), while two patients died. The additive effect of both good collaterals and reperfusion on outcome is shown Figure 2 and is substantiated by the predicted cumulative probabilities for clinical outcome (Fig. 3). The effect of successful reperfusion on outcome seemed to be greater than that of good collaterals with higher odds for successful reperfusion (Table 3), statistically being only a trend. In summary, we found reperfusion and collaterals to be independent predictors of clinical outcome, with no reciprocal effect modulation.

## Discussion

Our retrospective study in 93 patients with ischemic stroke in the anterior circulation treated with EVT, revealed the following independent predictors of favorable clinical outcome: successful reperfusion, younger age, good collaterals, female sex, and a good initial NCCT ASPECTS. CTA collateral status had the highest predictive value of all evaluated CT or CTA parameters. Outcome was best in patients with both good collaterals and successful reperfusion. All patients with poor collaterals who did not reperfuse died. Unlike previous studies which mostly used binary logistic regression models, we used proportional odds regression which permits full use of the ordinality of the outcome data and gives predictions for the whole range of the mRS (Scott et al. 1997; Sajobi et al. 2015). In addition, variable selection was data driven to allow for unbiased modeling.

Despite variations in scoring CT‐angiographic collaterals, their importance as a predictive factor in acute stroke patients has been acknowledged (McVerry et al. 2012), and has recently been confirmed for stroke patients treated with EVT (Galimanis et al. 2012; Nambiar et al. 2013; Seeta Ramaiah et al. 2014; Menon et al. 2015a). Previous studies indicated a high predictive value of successful reperfusion (Rha and Saver 2007) and pretreatment CT‐angiographic collateral status (Miteff et al. 2009; Lima et al. 2010) on clinical outcome. Others looked at the interaction of reperfusion and collaterals on final outcome in acute stroke patients treated with both IVT (Miteff et al. 2009) and EVT (Nambiar et al. 2013; Seeta Ramaiah et al. 2014) and yielded equivocal results. Some found a differential effect of reperfusion after EVT on clinical outcome depending on collateral status (Nambiar et al. 2013), others did not (Seeta Ramaiah et al. 2014). A compelling hypothesis in favor of an association is that good collaterals preserve the penumbra until reperfusion occurs (Miteff et al. 2009). In line with these findings, CTA‐collaterals were used as an imaging selector in a recent stroke trial demonstrating the superiority of EVT in combination with standard therapy compared to standard therapy alone (Demchuk et al. 2015; Goyal et al. 2015). Our analysis did not show a significant interaction of reperfusion and collaterals on outcome, that is, the effect of reperfusion on outcome seemed to be independent of the collaterals rated on CTA.

Currently, there is no reference standard for noninvasive imaging‐based selection criteria for EVT in patients with ischemic stroke (Menon et al. 2015b). In most institutions, NCCT and CTA constitute the imaging modalities of choice for treatment decision in patients with acute stroke. NCCT ASPECTS as a measure of infarct size is associated with functional outcome (Barber et al. 2000). CTA goes beyond the mere visualization of the vessel occlusion and provides additional parameters, which are proven outcome predictors: CTA‐SI ASPECTS (Bhatia et al. 2011), CBS (Puetz et al. 2008), and CTA‐collaterals (Schramm et al. 2002; Tan et al. 2007; Miteff et al. 2009).

In comparison to recent multicenter EVT trials, our patients had a lower median NCCT ASPECTS score of 7 compared to 9 in ESCAPE and SWIFT‐PRIME reflecting the relatively large proportion of patients with ICA occlusions in our cohort (Goyal et al. 2015; Saver et al. 2015). This might partially explain the lower rate of good outcome (mRS 0–2 in 31%) in our patients. Additionally, our reperfusion results were lower with mTICI 2b/3 in only 58% compared to 72.4% (ESCPAE) and 88% (SWIFT‐PRIME). This may have further reduced the odds for better clinical outcomes as a reperfusion result of mTICI >2b is prognostic for good clinical outcome (Yoo et al. 2013).

We could confirm that higher patient age reduces the prospects for a good clinical outcome (Castonguay et al. 2014). A short delay to initiation of treatment with IV tPA enhances the odds for a good outcome in acute stroke patients (Emberson et al. 2014). The same seems true for EVT where time to reperfusion governs clinical outcome (Khatri et al. 2014). However, in a large retrospective study (623 patients), time to treatment was a significant predictor only when ignoring the effect of pial collaterals (Galimanis et al. 2012). Correspondingly, time to endovascular treatment was only at the threshold of significance in our regression model which controlled for collateral status.

This study has limitations, mostly due to its retrospective design. Underpowered and unbalanced data (e.g. no patient with poor collaterals and without reperfusion had a good outcome) might have precluded detection of a significant interaction of reperfusion and collaterals as described before (Miteff et al. 2009; Nambiar et al. 2013). Due to a lack of angiographic data (not every patient received an angiogram of the contralateral ICA), we were unable to assess and compare the collaterals in DSA to those seen in CTA. The CT‐CS score as used in our study with values ranging from 0 to 3 was probably not an optimal differentiator concerning clinical outcome, as most of the patients were evaluated with a rather good collateral score of CT‐CS 2, but showing a broad spectrum of outcomes. Further refinement of the category CT‐CS 2 could provide better prediction of outcome. Due to a lack of comparative data, we dichotomized CT‐CS based on our own outcome evaluation.

We used single‐phase CT‐angiography which might overrate CBS and underrate CT‐CS compared to more time‐sensitive methods as multiphase CTA and 4D‐CTA (Frölich et al. 2013; Menon et al. 2015b). Although a promising method, CT perfusion was not included in our imaging protocol for stroke patients due to a lack of standardization of the method and as it adds time to image evaluation (Dani et al. 2011; Menon et al. 2015b). Finally, we used heterogeneous thrombectomy methods, which was due to the considerable evolution in treatment options during the study period. Small numbers precluded comparison of the treatment methods.

In conclusion, CTA‐collateral status was the strongest pretherapeutic predictor of clinical outcome in ischemic stroke patients treated with EVT. CTA‐collaterals are thus well suited for patient selection in EVT. However, in our study, the independent effect of reperfusion on outcome tended to be stronger than that of collaterals.

## Conflict of Interest

We declare that we have no conflict of interest and did not receive any external funding for the work.
